# Cigarette Smoke-Induced Cell Death Causes Persistent Olfactory Dysfunction in Aged Mice

**DOI:** 10.3389/fnagi.2018.00183

**Published:** 2018-06-13

**Authors:** Rumi Ueha, Satoshi Ueha, Kenji Kondo, Shu Kikuta, Tatsuya Yamasoba

**Affiliations:** ^1^Department of Otolaryngology and Head and Neck Surgery, Graduate School of Medicine, The University of Tokyo, Tokyo, Japan; ^2^Division of Molecular Regulation of Inflammatory and Immune Diseases Research Institute for Biomedical Sciences, Tokyo University of Science, Chiba, Japan

**Keywords:** cigarette smoking, aging, olfactory epithelium, olfactory receptor neuron, olfactory disfunction, inflammatory cytokines, apoptosis

## Abstract

**Introduction**: Exposure to cigarette smoke is a cause of olfactory dysfunction. We previously reported that in young mice, cigarette smoke damaged olfactory progenitors and decreased mature olfactory receptor neurons (ORNs), then, mature ORNs gradually recovered after smoking cessation. However, in aged populations, the target cells in ORNs by cigarette smoke, the underlying molecular mechanisms by which cigarette smoke impairs the regenerative ORNs, and the degree of ORN regeneration after smoking cessation remain unclear.

**Objectives**: To explore the effects of cigarette smoke on the ORN cell system using an aged mouse model of smoking, and to investigate the extent to which smoke-induced damage to ORNs recovers following cessation of exposure to cigarette smoke in aged mice.

**Methods**: We intranasally administered a cigarette smoke solution (CSS) to 16-month-old male mice over 24 days, then examined ORN existence, cell survival, changes of inflammatory cytokines in the olfactory epithelium (OE), and olfaction using histological analyses, gene analyses and olfactory habituation/dishabituation tests.

**Results**: CSS administration reduced the number of mature ORNs in the OE and induced olfactory dysfunction. These changes coincided with an increase in the number of apoptotic cells and *Tumor necrosis factor (TNF)* expression and a decrease in *Il6* expression. Notably, the reduction in mature ORNs did not recover even on day 28 after cessation of treatment with CSS, resulting in persistent olfactory dysfunction.

**Conclusion**: In aged mice, by increasing ORN death, CSS exposure could eventually overwhelm the regenerative capacity of the OE, resulting in continued reduction in the number of mature ORNs and olfactory dysfunction.

## Introduction

Olfaction is mediated by the olfactory system, which is composed of olfactory receptor neurons (ORNs) in the nasal cavity and the olfactory bulb in the forebrain (Su et al., 2009). ORNs have regenerative potential through the olfactory epithelial stem-cell system (Bermingham-McDonogh and Reh, 2011). Olfactory dysfunction can occur due to a variety of causes, such as aging, exposure to toxic chemicals, airway allergy, upper-airway viral infections, head trauma and neurodegenerative diseases (Weiler and Farbman, 1997; Doty, 2009; Ueha et al., 2014, 2018).

Age-related changes are well-known factors that affect olfaction. The elderly have a high incidence of olfactory impairment (Bihun and Percy, 1995) and recovery from olfactory dysfunction is decreased in aged populations (Walker et al., 1990). Aging-related olfactory dysfunction is associated with several histological changes in the olfactory neuroepithelium such as a thinning of the olfactory neuroepithelium (Weiler and Farbman, 1997; Watanabe et al., 2006; Kondo et al., 2009) and respiratory epithelial metaplasia (Paik et al., 1992; Nagano et al., 1997; Rosli et al., 1999). In humans, a part of the olfactory neuroepithelium in the elderly is converted into metaplastic respiratory epithelium and the metaplastic regions increase with age (Holbrook et al., 2011; Suzukawa et al., 2011). These morphological changes suggest that there is impairment in the regenerative function of ORNs in aged populations.

Cigarette smoking is a major cause of hyposmia and anosmia (Chen et al., 2003; Kushi et al., 2006; Katotomichelakis et al., 2007). The numerous chemical irritants contained in cigarette smoke trigger the expression of inflammatory mediators, such as interleukin (IL)-1β, IL-6, and tumor necrosis factor (TNF), in the respiratory tract (Nyunoya et al., 2014; Ueha et al., 2017). These mediators damage epithelial tissue and induce inflammatory responses, and a long history of cigarette smoking significantly increases the risk of various respiratory diseases (Hellermann et al., 2002; Nyunoya et al., 2014). In young adult populations, cigarette smoking decreases the thickness of the olfactory epithelium (OE; Kern et al., 2004) and increases apoptosis in the OE (Doty, 2009). In a previous study (Ueha et al., 2016a), we established a practical and reproducible model of cigarette smoking in which olfactory impairment developed after 20 doses of intranasal cigarette smoke solution (CSS) administration over 24 days using 8-week old male mice. In addition, we reported that exposure to CSS induced reduction in the number of mature ORNs and olfactory dysfunction by damaging the SOX2^+^ ORN progenitor population and increasing ORN death (Ueha et al., 2016a), while the ORN progenitor population and olfaction recovered following cessation of exposure to CSS (Ueha et al., 2016a). Moreover, we recently demonstrated that CSS impaired regeneration of ORNs by suppressing the development of immature ORNs from ORN progenitors, at least partly by reducing IGF-1 in the nasal mucosa (Ueha et al., 2016b).

Aging is linked to a higher risk of olfactory dysfunction, and smoking has also been associated with decreased olfaction in the elderly (Nicita-Mauro et al., 2008); thus, these two elements, aging and smoking, could have a synergistic negative impact on olfaction. However, in aged populations, the cellular and molecular mechanisms by which cigarette smoke disrupts the OE and olfaction remain largely unclear. We therefore hypothesized that in aged populations, cigarette smoke-induced inflammation may also disrupt the olfactory progenitor cell system and that complete recovery may not be possible.

In the present study, we explored the effects of cigarette smoke on the ORN cell system using an aged mouse model of smoking that involved administration of a CSS. We also investigated the extent to which CSS-induced damage to the ORNs recovers following cessation of exposure to CSS in aged mice, using histological analyses, olfactory habituation/dishabituation tests, and quantitative real-time polymerase chain reaction (qPCR) analyses.

## Materials and Methods

### Mice

C57BL/6 mice were purchased from Saitama Experimental Animals (Saitama, Japan). Sixteen-month-old mice were housed in a temperature-controlled environment under a 12-h light-dark cycle with access to food and water *ad libitum*. All animal experiments were conducted in accordance with institutional guidelines and with the approval of the Animal Care and Use Committee of the University of Tokyo (No. P14-086).

### Mouse Model of Smoking

**Figure 1:**
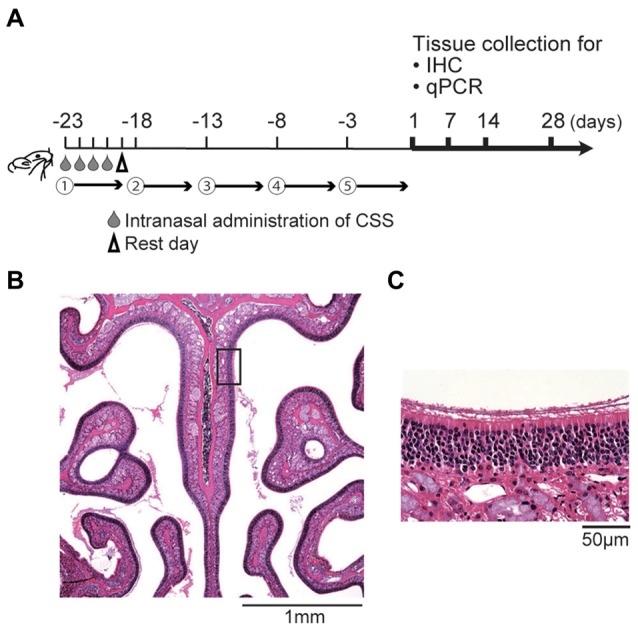
**(A)** Experimental timeline. Mice were intranasally administered a cigarette smoke solution (CSS) between days -23 and day 0 (five cycles, each including four daily doses of 20 μL/mouse and one rest day). Subsequently, the olfactory epithelium (OE) was collected for immunohistochemistry (IHC) and quantitative real-time polymerase chain reaction (qPCR) on the days indicated. **(B,C)** Representative images of hematoxylin and eosin stained sections of the OE from untreated mice (**B**, 40× magnification; **(C)** 400× magnification). The box in **(B)** indicates the region of the OE shown at higher magnification in **(C)**.

Using a CSS, we prepared the mouse model of cigarette smoking as previously reported (Ueha et al., 2016a). The CSS was produced by bubbling a stream of the smoke of Hi-Lite cigarettes (Japan Tobacco Inc., Tokyo, Japan) through saline (1 mL/cigarette) purchased from Cmic Bioresearch Center Co., Limited (Yamanashi, Japan; CSS, 20 μL/animal/time). The CSS was administered intranasally once a day on days −23 to −19. This cycle of four consecutive CSS administrations followed by 1 rest day was then repeated, with cycles 2–5 conducted over days −18 to −14, −13 to −9, −8 to −4, and −3 to 0, respectively (Figure 1). Control mice received saline intranasally according to the same schedule as CSS mice. CSS mice were sacrificed on days 1, 7, 14 and 28 after the final administration of CSS. Control mice were sacrificed on day 1 after the final administration of saline. Twelve mice were used for each CSS and control groups; six mice for histological analyses and six mice for gene analyses. Control mice were not assigned on days 7, 14 and 28 after the final administration of saline in accordance with our previous protocol (Ueha et al., 2016a), because of the limited number of aged mice.

### Tissue Preparation

The septal nasal mucosa was harvested on days 1, 7, 14 and 28 after the final intranasal administration of CSS for histological and qPCR analyses as previously described (Ueha et al., 2016a). Immediately after sacrificing the mice, the nasal cavities were gently irrigated with 4% paraformaldehyde in order to minimize mechanical damage to the OE. After decalcification, the tissues were dehydrated in a series of graded ethanol solutions, then embedded in paraffin.

### Antibody Staining

We stained tissues with the following anti-mouse primary antibodies: SOX2 (1:300 dilution; rabbit monoclonal, Abcam clone EPR3131; Abcam, Cambridge, MA, USA), GAP43 (1:1000 dilution; rabbit polyclonal, Novus #NB300-143B; Novus, Littleton, CO, USA), Ki67 (1:200 dilution; rabbit monoclonal, Novus #NB600-1252), OMP (1:8000 dilution, goat polyclonal, Wako, Tokyo, Japan), and cleaved capase-3 (1:300 dilution; rabbit polyclonal, Cell Signaling #9661; Cell Signaling, Danvers, MA, USA). SOX2 is a transcription factor that is widely expressed in stem cell populations, including neural stem cells, and plays a role in maintaining their undifferentiated state (Pevny and Placzek, 2005). In the OE, SOX2 is expressed by proliferating stem cells or progenitor cells in the basal layer and regulates homeostasis of the OE (Kawauchi et al., 2005, 2009; Guo et al., 2010; Bermingham-McDonogh and Reh, 2011; Ueha et al., 2014). SOX2 expression was also observed in sustentacular cells in the luminal layer, which lack progenitor activity, but these cells were excluded from our quantification of SOX2^+^ ORN progenitors. GAP43 is a growth-associated protein, and antibodies against GAP43 stain immature neurons. In the OE, GAP43 is expressed by immature ORNs (Katotomichelakis et al., 2007). OMP is an olfactory marker protein and is exclusively expressed in mature ORNs (Buiakova et al., 1996). The Ki67 protein is a cellular marker of proliferation and is strictly associated with cell proliferation (Starborg et al., 1996), and Ki67-positive cells are detected throughout the depth of the OE, mainly in the basal layer. On the other hand, caspases are crucial mediators of programmed cell death (apoptosis), and Cas3 is a frequently activated death protease, catalyzing the specific cleavage of many key cellular proteins (Porter and Janicke, 1999).

### Histological Analyses

As previously described (Ueha et al., 2014, 2016a), all samples were cut at the level of the anterior end of the olfactory bulb. Four-micrometer thick paraffin sections were deparaffinized in xylene and rehydrated in ethanol before immunostaining. For immunostaining, deparaffinized sections were treated with 3% hydrogen peroxide to block endogenous peroxidase activity and were incubated in Blocking One (Nacalai Tesque, Kyoto, Japan) to block non-specific immunoglobulin binding. Primary antibodies were detected using peroxidase-conjugated secondary antibodies and a diaminobenzidine (DAB) substrate. Three different microscope fields (dorsal, middle and ventral) of each bilateral septal OE were captured using a digital microscope camera (Keyence, Osaka, Japan, BZ-9000) with a 40× objective lens (Figure 1B). Analyses were restricted to the OE of the nasal septum to minimize variation between specimens (Figure 1C). The numbers of olfactory marker protein-positive (OMP^+^) ORNs was quantified by averaging the number of cells in each of the three microscopic fields between sections. The number of SOX2^+^ ORN progenitors, GAP43^+^ immature ORNs, Ki67^+^ cells, and cleaved Cas3^+^ apoptotic cells per mm of basal layer length were manually counted using digital imaging software (Photoshop CS6 Adobe, San Jose, CA, USA), in a blinded manner for each of the three different fields.

### Quantitative Real-Time Polymerase Chain Reaction

Total RNA was isolated from the septal nasal mucosa using TRIzol reagent (Life Technologies, Gaithersburg, MD, USA) on days 1, 7, 14 and 28 after the final intranasal administration of the CSS, and then converted to cDNA using the ReverTra Ace qPCR RT Master Mix with gDNA Remover (Toyobo, Osaka, Japan) according to the manufacturer’s instructions. qPCR analysis was performed using the THUNDERBIRD Probe qPCR Mix or THUNDERBIRD SYBR qPCR Mix (Toyobo) and an ABI 7500 sequence detector system (Life Technologies). The gene-specific primers and probes used were: Rps3 as endogenous control (Life Technologies assay number Mm00656272_m1); Il1b (forward 5′-AGGCAGGCAGTATCACTCATTGT-3′, reverse 5′-CGTCACACACCAGCAGGTTATC-3′); and Tnf (forward 5′-TGTGCCTCAGCCTCTTCTC-3′, reverse 5′-GAGCCCATTTGGGAACTTCT-3′); and Il6 (forward 5′-CTGCAAGAGACTTCCATCCAGTT-3′, reverse 5′-AGGTCTGTTGGGAGTGGTATCC-3′). The expression levels of each gene were normalized to the level of Rps3 expression for each sample.

### Behavioral Testing to Evaluate Olfactory Function

Olfactory sensitivity was evaluated with an olfactory habituation/dishabituation test that was performed on days 7, 14 and 28 after the final intranasal administration of CSS, following a previous procedure (Ueha et al., 2016a). Mice were allowed to acclimatize in a clean plastic cage for 30 min, then presented with a piece of filter paper soaked in odorless mineral oil for 3 min. This procedure was repeated a total of four times at 1-min intervals. However, for the fourth exposure, the filter paper was soaked in propyl propionate instead of mineral oil. Throughout each test, mouse behavior was recorded using a digital video camera. The nose of the mouse being within 1 mm of the filter paper was considered to represent “investigative behavior.” The duration of investigative behavior was compared between the third and fourth exposures. Mice with normal olfaction display gradually reduced durations of investigative behavior from the first to third exposures (habituation) but display reinstatement of investigative behavior when an odor is presented with the filter paper. A lack of reinstatement indicates reduced or absent olfactory sensitivity.

### Statistical Analysis

Statistical comparisons between groups or time-points were performed by paired Student’s *t*-test or by one-way ANOVA (as indicated in the figure legends) using GraphPad Prism software (version 6.0; GraphPad Software Inc., San Diego, CA, USA). qPCR data were subjected to logarithmic transformation prior to analysis. *P*-values < 0.05 were considered to be statistically significant.

## Results

### Impairment of Olfactory Receptor Neurons Caused by CSS Exposure Is Prolonged in Aged Mice

**Figure 2:**
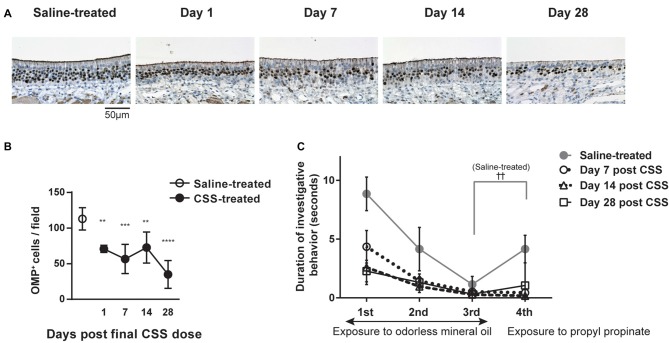
**(A)** Immunohistochemical staining (brown) of olfactory marker protein-positive (OMP^+^) cells in the OE 1 day after the final dose of CSS. Mice received 20 doses (five cycles) of CSS. Data represent mean ± SEM (*n* = 6). **P < 0.01 (Mann-Whitney U test). **(A)** Representative images of immunohistochemical staining (brown) of OMP^+^ cells in the OE in saline-treated mice and on various days following the final CSS dose (*n* = 6). **(B)** Number of OMP^+^ olfactory receptor neurons (ORNs) per field in saline or CSS-treated mice. Data represent means ± SEM (*n* = 6). **P < 0.01; ***P < 0.001; ****P < 0.0001 compared with saline-treated mice (one-way ANOVA). **(C)** Olfactory habituation/dishabituation test. Mice were presented with a piece of filter paper soaked in odorless mineral oil three times for 3 min, at 1-min intervals. For the fourth exposure, the filter paper was soaked in the odorant propyl propionate instead of mineral oil. Data represent means ± SEM (*n* = 6). ^††^*P* < 0.01, third exposure compared with the fourth exposure (paired Student’s *t*-test). No significant differences were detected between the third and fourth exposures for CSS-treated mice on days 1–28, suggesting a decrease in olfactory sensitivity. CSS, cigarette smoke solution; OMP+, olfactory marker protein-positive.

We first examined the effect of continuous intranasal administration of CSS on ORNs in the aged mice (see experimental timeline in Figure 1). In the previous study using 8-week old adult mice, with the same time course (Ueha et al., 2016a), exposure to CSS induced reduction in numbers of mature ORNs and the number of mature ORNs recovered following cessation of exposure to CSS. Immunohistochemical staining revealed that OMP^+^ mature ORNs formed thick layers in the OE of saline-treated mice (Figure 2A). In contrast, the number of OMP^+^ cells in the mice that received 20 doses (five cycles) of CSS administration was approximately 60% less than the numbers observed in saline-treated mice 1 day after the final treatment (Figures 2A,B). We next examined whether the OMP^+^ mature ORN population would recover after cessation of CSS administration. A time course analysis revealed that the reduction in OMP^+^ ORN numbers continued even after cessation of CSS administration, and that the reduction became more severe on day 28 after the final intranasal administration of CSS (Figures 2A,B). This phenomenon is characteristic of aged mice and is not observed in young adult mice (Ueha et al., 2016a). The prolonged impairment of the OE suggests that CSS-induced damage to the OE is not simply due to acute damage to mature ORNs, but rather that additional long-term factors are involved in impairment of ORNs by CSS.

### Olfactory Dysfunction Is Induced by CSS Exposure and Continues in Aged Mice

We examined whether the CSS-induced reduction in OMP^+^ mature ORN numbers is associated with impaired olfaction by using a habituation/dishabituation test to evaluate olfactory sensitivity. In this test, propyl propionate, which is predominantly perceived by the OE (Kanaya et al., 2014), was used as the odorant. Saline-treated mice displayed decreasing durations of investigative behavior when repeatedly exposed to a piece of filter paper soaked with odorless mineral oil, which is consistent with habituation (Figure 2C). In saline-treated mice, when the filter paper was soaked with propyl propionate on the fourth exposure, the duration of investigative behavior was significantly longer than that on the third trial, suggesting that the mice were capable of smelling the odorant (dishabituation). In contrast, while CSS-treated mice displayed a similar pattern of habituation as saline-treated mice, there was no significant difference in the duration of investigative behavior between the third and fourth trials on day 7 after the final CSS administration, suggesting a decrease in olfactory sensitivity in the CSS-treated mice. Surprisingly, the loss of olfactory sensitivity continued even on days 14 and 28 after the final CSS administration, while in young adult mice, olfaction recovered following cessation of exposure to CSS (Ueha et al., 2016a). These results show that olfactory function is impaired by CSS administration in aged mice and that olfactory function does not recover by day 28 after the final CSS administration.

### CSS Exposure Induces Cell-Death of ORNs and Considerably Impacts ORN Numbers

**Figure 3:**
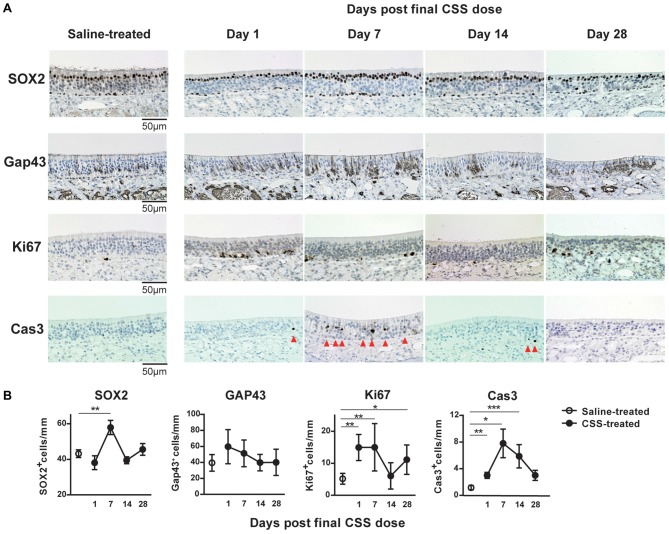
**(A)** Representative images of immunohistological staining (brown) of SOX2^+^ olfactory receptor neurons (ORN) progenitor cells, Gap43^+^ immature ORNs, Ki67^+^ proliferating cells and cleaved caspase-3^+^ (Cas3^+^) apoptotic cells. Tissue sections were counterstained with the nuclear dye hematoxylin (blue). Arrowheads indicate Cas3^+^ apoptotic cells in the OE (*n* = 6). **(B)** Numbers of SOX2^+^ ORN progenitors and Ki67^+^ actively proliferating cells per mm of the basal layer, and Gap43^+^ immature ORNs and Cas3^+^ apoptotic cells per mm of the OE in saline or CSS-treated mice. Data represent means ± SEM (*n* = 6). *P < 0.05; **P < 0.01; ***P < 0.001 compared with saline-treated mice (one-way ANOVA).

The similarity in the insufficiency of recovery of OMP^+^ ORN numbers and olfaction following cessation of CSS administration suggested that damage to the OMP^+^ ORNs may underlie CSS-induced hyposmia and anosmia. Moreover, these insufficiencies lasted at least until day 28 after the final CSS administration. As the number of OMP^+^ mature ORNs is determined by the balance between the cell death of ORNs and the regeneration of ORNs from ORN progenitors, we next investigated the effects of cigarette smoke on the number of ORN progenitors, immature ORNs and dividing cells, and on cell death in the aged mice, by examining SOX2 expression, GAP43, Ki67, and cleaved caspase-3. Ki67^+^ cells detected throughout the depth of the epithelium (mainly in the basal layer of the OE; Suzukawa et al., 2011). In saline-treated mice, a large number of SOX2^+^ ORN progenitors and a small number of Ki67^+^ proliferating cells were detected in the basal layer (Figure 3A). Although SOX2 expression was also observed in sustentacular cells in the luminal layer without progenitor activity, these cells were excluded from our quantification of SOX2^+^ ORN progenitors. GAP43^+^ immature ORNs were observed in the area above the basal layer. Cleaved Cas3^+^ apoptotic cells were scarcely detected in the OE. In CSS-treated mice, although the number of SOX2^+^ ORN progenitors in the basal layer did not show any changes compared to that in saline-treated mice on day 1 after final CSS administration, it was significantly elevated on day 7 and had returned to saline treated-mouse levels by day 14 (Figure 3B). The number of Ki67^+^ proliferating cells in the basal layer changed with a time course similar to that of SOX2^+^ ORN progenitors except of that on day 1 after final CSS administration. No significant differences in the number of GAP43^+^ immature ORNs were detected between the saline-treated mice and the CSS-treated mice over the time points examined. Cleaved Cas3^+^ apoptotic cells were more frequent on day 7 and somewhat more frequent on days 1 and 14 after final CSS administration.

Given that OMP^+^ ORNs are maintained by the continuous proliferation and subsequent maturation of SOX2^+^ ORN progenitors in the steady-state, these results suggest that the CSS-induced death of ORNs overwhelms increased proliferation of ORN progenitors in aged mice exposed to CSS and that the CSS-induced death of ORNs has a considerable impact on ORN numbers and olfaction.

### CSS Administration Does Not Induce Increases in Inflammatory Cytokine Expressions in the Olfactory Epithelium of Aged Mice

Finally, because inflammatory cytokines influence the proliferation and differentiation of neural stem and progenitor cells, we examined the possible involvement of inflammatory cytokines in the CSS-induced disruption of the ORN progenitor cell population. Generally, aging is associated with increased inflammatory activity reflected by increased circulating levels of proinflammatory cytokines, such as IL-1β and TNF, and hence the levels of inflammatory cytokines in the aged tissue are higher than those in the young (Pedersen et al., 2000). In addition, smoking is reported to upregulate proinflammatory cytokines (Ueha et al., 2016a).

**Figure 4:**
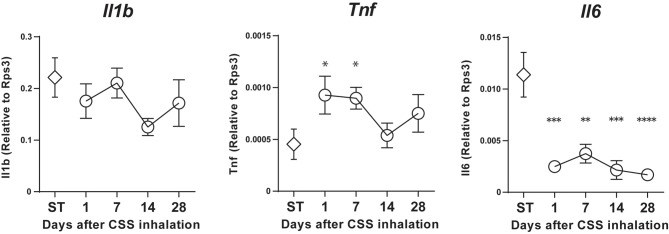
*Il1b*, Tumor necrosis factor (TNF) and *Il6* mRNA expression in the nasal mucosa was quantified by quantitative real-time polymerase chain reaction (qPCR) in saline (ST) or CSS-treated mice and calculated relative to the expression of the endogenous control gene *Rps3*. Data represent means ± SEM (*n* = 6). *P < 0.05; **P < 0.01; ***P < 0.001; ****P < 0.0001 (one-way ANOVA).

Our time course analyses revealed that *Tnf* expression was significantly increased on days 1 and 7 after final CSS administration compared to the saline-treated group. However, *Il1b* mRNA expression did not show any significant changes in the nasal mucosa on days 1–28 after final CSS administration, relative to expression in saline-treated mice, and even then, the mRNA expression of *Il1b* was maintained at an age-related high level. Unexpectedly, *Il6* expression was significantly decreased contrary to expectations on days 1–28 after final CSS administration compared to the saline-treated group (Figure 4).

## Discussion

In the present study, we demonstrated that long-term CSS administration decreased OMP^+^ mature ORNs in aged mice and it is suggested that cigarette smoke can disrupt the balance between proliferation and apoptosis in the OE of aged populations. The reduction in mature ORN numbers could be associated with olfactory dysfunction. Even after ceasing CSS administration, deterioration of ORN numbers further proceeded at least for 28 days; as a result, aged mice might not react to odorants even at 28 days after the last CSS administration, whereas olfactory impairment was transient in young adult mice (Ueha et al., 2016a).

In young adult mice, CSS induced decrease in the numbers of ORN progenitors and proliferating cells and increase in apoptotic ORNs on day 1 after then final CSS administration. Therefore, these changes in cell dynamics appeared to be involved in the CSS-induced decrease in the ORN population (Ueha et al., 2016a). However, in the aged mice, time course analyses also revealed that the number of SOX2^+^ ORN progenitors was not statistically significantly changed after CSS exposure but mature ORN numbers declined compared with the young mice (Ueha et al., 2016a). The reason for this can be the milder decrease in the numbers of Ki67^+^ proliferating cells and the more continuing elevation in apoptotic ORNs in comparison with the young mice. Regarding the increase in proliferating cells in aged mice on day 1 after the final CSS administration, it could be attributable to that proliferated ORNs possibly remain immature, that apoptosis in ORNs is predominant, and that ORN proliferating cells possibly appear earlier than in young mice, as reflected in the tissue repair response. Taken together, the increase in apoptotic ORNs may be a main cause of decreased ORN populations in the aged mice treated with CSS.

The characteristics of the response of aged OE to CSS may be attributed to the persistent inflammation of aged tissue. Generally, in aged populations, age-associated buildup of inflammatory cytokines (TNF, IL-1β and IL-6) within tissues is noted (Chaker et al., 2015; Ueha et al., 2018), and this elevation in inflammatory cytokines may result in a sustained state of chronic inflammation, known as the senescence-associated secretory phenotype, in aged populations. In the present study, the expression levels of *Il1b* and *Il6* in the saline-treated aged mice were higher than those in the saline-treated young mice in the previous study (Ueha et al., 2016a). An increase of *Tnf* expression in the CSS-treated aged mice continued for at least 7 days after the last CSS administration. Our results suggest that upregulation of *Tnf* expression may increase cell apoptosis in the OE after CSS exposure and is possibly involved in a persistent decline of mature ORNs and olfaction in aged mice (Suzuki and Farbman, 2000).

The exact mechanism by which cigarette smoke disrupts the balance of the OE in aged populations remains unclear, but it seems likely that cigarette smoke could damage ORNs by inducing inflammation and increasing apoptosis in the OE, especially in aged mice, similar to the way in which cigarette smoke toxicity in the lungs is largely mediated by inflammation and immunotoxicity (Hellermann et al., 2002; Nyunoya et al., 2014). Our observation that CSS induces expression of *Tnf* is consistent with this theory. Considering that elevated levels of *Tnf* and decreased levels of *Il6* expression persisted for at least 7 and 28 days after final CSS administration, respectively, and that it coincided with the increased numbers of apoptotic cells in the OE, these inflammatory cytokines may positively or negatively regulate the suppression of ORN maturation from their progenitors or induce apoptosis.

Regarding the role of TNF in the OE, it has been reported that TNF induces inflammation and apoptosis in the OE, while also suppressing the normal regenerative replacement mechanism and inhibiting the ORN progenitors, resulting in thinning of the olfactory neuron layer and a progressive loss of olfactory function (Suzuki and Farbman, 2000; Turner et al., 2010a,b). Thus, our findings are consistent with these theories. The roles of IL-6 in the OE have not been well elucidated. On one hand, IL-6 can protect cells from injurious stimuli via the inhibition of apoptosis (Teague et al., 1997), suppresses the expression of SOX2 (Yoon et al., 2014), and is involved in tissue repair and supports cell proliferation (Liechty et al., 2000). On the other hand, IL-6 is supposed to suppress OE regeneration following injury and is considered to play an important role in the development of hyposmia (Xie et al., 2013). Considering the coincident decrease in Il6 expression and increase in apoptotic cells after CSS exposure in aged mice, Il6 possibly plays a protective role in CSS-treated aged mice.

In the present study, we focused on the effects of CSS exposure on the OE of aged mice. However, olfactory dysfunction can also be induced by damage to the olfactory bulb in the forebrain. Accordingly, damage to the olfactory bulb could also be responsible for some olfactory dysfunction. Future neurophysiological studies that quantify signal transduction from the ORNs to the olfactory bulb are necessary to fully elucidate the pathogenesis of cigarette smoke-induced olfactory dysfunction in aged populations. Moreover, regarding the behavioral testing in the present study, limitations should be taken into account. Considering that there were the differences in the duration of investigative behavior between control mice and CSS-exposure mice on the first exposure, the results from the behavioral testing could be influenced by factors such as curiosity, motivation, and motor function, which might be caused by CSS exposure.

## Conclusion

We demonstrated that CSS exposure reduces the number of mature ORNs and olfactory dysfunction by increasing ORN death in the OE of aged mice, which eventually overwhelms the regenerative capacity of the epithelium. Moreover, the ORN population and olfaction had not recovered 28 days after cessation of exposure to CSS. These findings provide a basis of the mechanisms underlying cigarette smoke-induced damage to ORNs in aged populations and therapeutic clues for the treatment of cigarette smoke-induced olfactory dysfunction in aged populations by TNF suppression and apoptosis inhibition.

## Author Contributions

RU, SU, KK and TY developed the concept, designed and performed the experiments and analyzed the data. SK performed the experiments and analyzed the data. All authors contributed to interpretation of the data and writing of the manuscript.

## Conflict of Interest Statement

The authors declare that the research was conducted in the absence of any commercial or financial relationships that could be construed as a potential conflict of interest.

## Abbreviations

CSS: cigarette smoke solution
OE: olfactory epithelium
OMP^+^: olfactory marker protein-positive
ORN: olfactory receptor neuron.

## References

[B1] Bermingham-McDonogh O. Reh T. A. (2011). Regulated reprogramming in the regeneration of sensory receptor cells. Neuron 71, 389–405. 10.1016/j.neuron.2011.07.015 21835338 PMC4403668

[B2] Bihun C. G. Percy D. H. (1995). Morphologic changes in the nasal cavity associated with sialodacryoadenitis virus infection in the Wistar rat. Vet. Pathol. 32, 1–10. 10.1177/030098589503200101 7725592

[B3] Buiakova O. I. Baker H. Scott J. W. Farbman A. Kream R. Grillo M.. (1996). Olfactory marker protein (OMP) gene deletion causes altered physiological activity of olfactory sensory neurons. Proc. Natl. Acad. Sci. U S A 93, 9858–9863. 10.1073/pnas.93.18.9858 8790421 PMC38519

[B4] Chaker Z. Aid S. Berry H. Holzenberger M. (2015). Suppression of IGF-I signals in neural stem cells enhances neurogenesis and olfactory function during aging. Aging Cell 14, 847–856. 10.1111/acel.12365 26219530 PMC4568972

[B5] Chen Y. Dales R. Lin M. (2003). The epidemiology of chronic rhinosinusitis in Canadians. Laryngoscope 113, 1199–1205. 10.1097/00005537-200307000-00016 12838019

[B6] Doty R. L. (2009). The olfactory system and its disorders. Semin. Neurol. 29, 74–81. 10.1055/s-0028-1124025 19214935

[B7] Guo Z. Packard A. Krolewski R. C. Harris M. T. Manglapus G. L. Schwob J. E. (2010). Expression of pax6 and sox2 in adult olfactory epithelium. J. Comp. Neurol. 518, 4395–4418. 10.1002/cne.22463 20852734 PMC2940252

[B8] Hellermann G. R. Nagy S. B. Kong X. Lockey R. F. Mohapatra S. S. (2002). Mechanism of cigarette smoke condensate-induced acute inflammatory response in human bronchial epithelial cells. Respir. Res. 3:22. 10.1186/rr172 12204101 PMC150508

[B9] Holbrook E. H. Wu E. Curry W. T. Lin D. T. Schwob J. E. (2011). Immunohistochemical characterization of human olfactory tissue. Laryngoscope 121, 1687–1701. 10.1002/lary.21856 21792956 PMC3181071

[B10] Kanaya K. Kondo K. Suzukawa K. Sakamoto T. Kikuta S. Okada K.. (2014). Innate immune responses and neuroepithelial degeneration and regeneration in the mouse olfactory mucosa induced by intranasal administration of Poly(I:C). Cell Tissue Res. 357, 279–299. 10.1007/s00441-014-1848-2 24744264 PMC4077259

[B11] Katotomichelakis M. Balatsouras D. Tripsianis G. Davris S. Maroudias N. Danielides V.. (2007). The effect of smoking on the olfactory function. Rhinology 45, 273–280. 18085020

[B12] Kawauchi S. Kim J. Santos R. Wu H. H. Lander A. D. Calof A. L. (2009). Foxg1 promotes olfactory neurogenesis by antagonizing Gdf11. Development 136, 1453–1464. 10.1242/dev.034967 19297409 PMC2674256

[B13] Kawauchi S. Shou J. Santos R. Hébert J. M. McConnell S. K. Mason I.. (2005). Fgf8 expression defines a morphogenetic center required for olfactory neurogenesis and nasal cavity development in the mouse. Development 132, 5211–5223. 10.1242/dev.02143 16267092

[B14] Kern R. C. Conley D. B. Haines G. K. III. Robinson A. M. (2004). Pathology of the olfactory mucosa: implications for the treatment of olfactory dysfunction. Laryngoscope 114, 279–285. 10.1097/00005537-200402000-00018 14755203

[B15] Kondo K. Watanabe K. Sakamoto T. Suzukawa K. Nibu K. Kaga K.. (2009). Distribution and severity of spontaneous lesions in the neuroepithelium and Bowman’s glands in mouse olfactory mucosa: age-related progression. Cell Tissue Res. 335, 489–503. 10.1007/s00441-008-0739-9 19142664

[B16] Kushi L. H. Byers T. Doyle C. Bandera E. V. McCullough M. McTiernan A.. (2006). American cancer society guidelines on nutrition and physical activity for cancer prevention: reducing the risk of cancer with healthy food choices and physical activity. CA Cancer J. Clin. 56, 254–281; quiz 313–254. 10.3322/canjclin.56.5.254 17005596

[B17] Liechty K. W. Adzick N. S. Crombleholme T. M. (2000). Diminished interleukin 6 (IL-6) production during scarless human fetal wound repair. Cytokine 12, 671–676. 10.1006/cyto.1999.0598 10843743

[B18] Nagano K. Katagiri T. Aiso S. Senoh H. Sakura Y. Takeuchi T. (1997). Spontaneous lesions of nasal cavity in aging F344 rats and BDF1 mice. Exp. Toxicol. Pathol. 49, 97–104. 10.1016/s0940-2993(97)80077-2 9085083

[B19] Nicita-Mauro V. Lo Balbo C. Mento A. Nicita-Mauro C. Maltese G. Basile G. (2008). Smoking, aging and the centenarians. Exp. Gerontol. 43, 95–101. 10.1016/j.exger.2007.06.011 17686596

[B20] Nyunoya T. Mebratu Y. Contreras A. Delgado M. Chand H. S. Tesfaigzi Y. (2014). Molecular processes that drive cigarette smoke-induced epithelial cell fate of the lung. Am. J. Respir. Cell Mol. Biol. 50, 471–482. 10.1165/rcmb.2013-0348TR 24111585 PMC4068939

[B21] Paik S. I. Lehman M. N. Seiden A. M. Duncan H. J. Smith D. V. (1992). Human olfactory biopsy. The influence of age and receptor distribution. Arch. Otolaryngol. Head Neck Surg. 118, 731–738. 10.1001/archotol.1992.01880070061012 1627295

[B22] Pedersen B. K. Bruunsgaard H. Ostrowski K. Krabbe K. Hansen H. Krzywkowski K.. (2000). Cytokines in aging and exercise. Int. J. Sports Med. 21, S4–S9. 10.1055/s-2000-1444 10893017

[B23] Pevny L. Placzek M. (2005). SOX genes and neural progenitor identity. Curr. Opin. Neurobiol. 15, 7–13. 10.1016/j.conb.2005.01.016 15721738

[B24] Porter A. G. Janicke R. U. (1999). Emerging roles of caspase-3 in apoptosis. Cell Death Differ. 6, 99–104. 10.1038/sj.cdd.4400476 10200555

[B25] Rosli Y. Breckenridge L. J. Smith R. A. (1999). An ultrastructural study of age-related changes in mouse olfactory epithelium. J. Electron Microsc. 48, 77–84. 10.1093/oxfordjournals.jmicro.a023653 10101872

[B26] Starborg M. Gell K. Brundell E. Höög C. (1996). The murine Ki-67 cell proliferation antigen accumulates in the nucleolar and heterochromatic regions of interphase cells and at the periphery of the mitotic chromosomes in a process essential for cell cycle progression. J. Cell Sci. 109, 143–153. 8834799 10.1242/jcs.109.1.143

[B27] Su C. Y. Menuz K. Carlson J. R. (2009). Olfactory perception: receptors, cells, and circuits. Cell 139, 45–59. 10.1016/j.cell.2009.09.015 19804753 PMC2765334

[B28] Suzukawa K. Kondo K. Kanaya K. Sakamoto T. Watanabe K. Ushio M.. (2011). Age-related changes of the regeneration mode in the mouse peripheral olfactory system following olfactotoxic drug methimazole-induced damage. J. Comp. Neurol. 519, 2154–2174. 10.1002/cne.22611 21452219

[B29] Suzuki Y. Farbman A. I. (2000). Tumor necrosis factor-α-induced apoptosis in olfactory epithelium *in vitro*: possible roles of caspase 1 (ICE), caspase 2 (ICH-1) and caspase 3 (CPP32). Exp. Neurol. 165, 35–45. 10.1006/exnr.2000.7465 10964483

[B30] Teague T. K. Marrack P. Kappler J. W. Vella A. T. (1997). IL-6 rescues resting mouse T cells from apoptosis. J. Immunol. 158, 5791–5796. 9190930

[B31] Turner J. H. Liang K. L. May L. Lane A. P. (2010a). Tumor necrosis factor α inhibits olfactory regeneration in a transgenic model of chronic rhinosinusitis-associated olfactory loss. Am. J. Rhinol. Allergy 24, 336–340. 10.2500/ajra.2010.24.3498 21243089 PMC3021182

[B32] Turner J. H. May L. Reed R. R. Lane A. P. (2010b). Reversible loss of neuronal marker protein expression in a transgenic mouse model for sinusitis-associated olfactory dysfunction. Am. J. Rhinol. Allergy 24, 192–196. 10.2500/ajra.2010.24.3460 20537285 PMC3021955

[B33] Ueha R. Mukherjee S. Ueha S. de Almeida Nagata D. E. Sakamoto T. Kondo K.. (2014). Viral disruption of olfactory progenitors is exacerbated in allergic mice. Int. Immunopharmacol. 22, 242–247. 10.1016/j.intimp.2014.06.034 24998164 PMC4129161

[B34] Ueha R. Shichino S. Ueha S. Kondo K. Kikuta S. Nishijima H.. (2018). Reduction of proliferating olfactory cells and low expression of extracellular matrix genes are hallmarks of the aged olfactory mucosa. Front. Aging Neurosci. 10:86. 10.3389/fnagi.2018.00086 29636678 PMC5880952

[B35] Ueha R. Ueha S. Kondo K. Nito T. Fujimaki Y. Nishijima H.. (2017). Laryngeal mucus hypersecretion is exacerbated after smoking cessation and ameliorated by glucocorticoid administration. Toxicol. Lett. 265, 140–146. 10.1016/j.toxlet.2016.11.023 27916735

[B36] Ueha R. Ueha S. Kondo K. Sakamoto T. Kikuta S. Kanaya K.. (2016a). Damage to olfactory progenitor cells is involved in cigarette smoke-induced olfactory dysfunction in mice. Am. J. Pathol. 186, 579–586. 10.1016/j.ajpath.2015.11.009 26806086

[B37] Ueha R. Ueha S. Sakamoto T. Kanaya K. Suzukawa K. Nishijima H.. (2016b). Cigarette smoke delays regeneration of the olfactory epithelium in mice. Neurotox. Res. 30, 213–224. 10.1007/s12640-016-9617-5 27003941

[B38] Walker D. G. Breipohl W. Simon-Taha A. Lincoln D. Lobie P. E. Garcia Aragon J. (1990). Cell dynamics and maturation within the olfactory epithelium proper of the mouse—a morphometric analysis. Chem. Senses 15, 741–753. 10.1093/chemse/15.6.741

[B39] Watanabe K. Kondo K. Takeuchi N. Nibu K. Kaga K. (2006). Age-related changes in cell density and the proliferation rate of olfactory ensheathing cells in the lamina propria of postnatal mouse olfactory mucosa. Brain Res. 1116, 82–92. 10.1016/j.brainres.2006.07.124 16952341

[B40] Weiler E. Farbman A. I. (1997). Proliferation in the rat olfactory epithelium: age-dependent changes. J. Neurosci. 17, 3610–3622. 10.1523/jneurosci.17-10-03610.1997 9133384 PMC6573682

[B41] Xie F. Fang C. Schnittke N. Schwob J. E. Ding X. (2013). Mechanisms of permanent loss of olfactory receptor neurons induced by the herbicide 2,6-dichlorobenzonitrile: effects on stem cells and noninvolvement of acute induction of the inflammatory cytokine IL-6. Toxicol. Appl. Pharmacol. 272, 598–607. 10.1016/j.taap.2013.07.020 23921153 PMC3805741

[B42] Yoon D. S. Kim Y. H. Lee S. Lee K. M. Park K. H. Jang Y.. (2014). Interleukin-6 induces the lineage commitment of bone marrow-derived mesenchymal multipotent cells through down-regulation of Sox2 by osteogenic transcription factors. FASEB J. 28, 3273–3286. 10.1096/fj.13-248567 24719354

